# Red seaweed extracts reduce methane production by altering rumen fermentation and microbial composition *in vitro*

**DOI:** 10.3389/fvets.2022.985824

**Published:** 2022-11-16

**Authors:** Youyoung Choi, Shin Ja Lee, Hyun Sang Kim, Jun Sik Eom, Seong Uk Jo, Le Luo Guan, Tansol Park, Jakyeom Seo, Yookyung Lee, Dongryeoul Bae, Sung Sill Lee

**Affiliations:** ^1^Division of Applied Life Science (BK21), Gyeongsang National University, Jinju, South Korea; ^2^Institute of Agriculture & Life Science (IALS), Gyeongsang National University, Jinju, South Korea; ^3^Institute of Agriculture and Life Science & University-Centered Labs, Gyeongsang National University, Jinju, South Korea; ^4^Department of Agricultural, Food and Nutritional Science, University of Alberta, Edmonton, AB, Canada; ^5^Department of Animal Science and Technology, Chung-Ang University, Anseong, South Korea; ^6^Department of Animal Science, Life and Industry Convergence Research Institute, Pusan National University, Miryang, South Korea; ^7^Animal Nutrition and Physiology Team, National Institute of Animal Science, Rural Development of Administration (RDA), Jeonju, South Korea; ^8^College of Pharmacy and Research Institute of Pharmaceutical Science, PMBBRC, Gyeongsang National University, Jinju, South Korea

**Keywords:** *Asparagopsis*, seaweed, *in vitro*, methane, metataxonomic, rumen microbiota

## Abstract

A series of *in vitro* batch culture incubations were carried out to investigate changes in rumen fermentation characteristics, methane (CH_4_) production, and microbial composition in response to supplementation with five different red seaweed species (*Amphiroa anceps*, AANC; *Asparagopsis taxiformis*, ATAX; *Chondracanthus tenellus*, CTEN; *Grateloupia elliptica*, GELL; and *Gracilaria parvispora*, GPAR). Prior to the incubations, the total flavonoid and polyphenol content of the red seaweed extracts was quantified. The incubated substrate consisted of timothy hay and corn grain [60:40 dry matter (DM) basis]. Treatments were substrate mixtures without seaweed extract (CON) or substrate mixtures supplemented with 0.25 mg/mL of red seaweed extract. Samples were incubated for 6, 12, 24, 36, and 48 h. Each sample was incubated in triplicates in three separate runs. *In vitro* DM degradability, fermentation parameters (i.e., pH, volatile fatty acids, and ammonia nitrogen), total gas production, and CH_4_ production were analyzed for all time points. Microbial composition was analyzed using 16S rRNA amplicon sequencing after 24 h of incubation. The highest CH_4_ reduction (mL/g DM, mL/g digested DM, and % of total gas production) was observed in ATAX (51.3, 50.1, and 51.5%, respectively, compared to CON; *P* < 0.001) after 12 h of incubation. The other red seaweed extracts reduced the CH_4_ production (mL/g DM; *P* < 0.001) in the range of 4.6–35.0% compared to CON after 24 h of incubation. After 24 h of incubation, supplementation with red seaweed extracts tended to increase the molar proportion of propionate (*P* = 0.057) and decreased the acetate to propionate ratio (*P* = 0.033) compared to the CON. Abundances of the genus *Methanobrevibacter* and total methanogens were reduced (*P* = 0.050 and *P* = 0.016) by red seaweed extract supplementation. The linear discriminant analysis effect size (*P* < 0.05, LDA ≥ 2.0) showed that UG Succinivibrionaceae, *Anaeroplasma*, and UG Ruminococcaceae, which are associated with higher propionate production, starch degradation, and amylase activity were relatively more abundant in red seaweed extracts than in the CON. Our results suggest that supplementation with red seaweed extracts altered the microbiota, leading to the acceleration of propionate production and reduction in CH_4_ production.

## Introduction

Recognizing the urgent need to address climate change, most nations have set a goal of achieving net-zero greenhouse gas (GHG) emissions by the second half of the twenty-first century (1). Methane (CH_4_) is a major source of atmospheric GHG (2). For example, CH_4_ emissions from the enteric fermentation of ruminants contribute to approximately 37.3% of the total GHG emissions across the agricultural sector in Korea (3). Methane produced by ruminants is not only related to climate change but also associated with a loss of energy (2–12% gross energy intake) of the host animals (4). Energy saved because of a decrease in CH_4_ production could potentially be available for animal performance and improve the efficiency of production (i.e., meat, milk, and wool) (5).

A variety of feed additives have been used to reduce CH_4_ production by modulating microbial methanogenesis within the rumen (ionophores, nitrate, etc.). Seaweeds (also known as red, green, or brown macroalgae) have been shown to be some of the most promising additives for this purpose. The global production of seaweeds reached 32.4 million tons in 2018, and seaweeds are currently harvested in approximately 50 countries (6, 7). Seaweeds are mainly used for direct human consumption in Asian countries, they have been used as a feed ingredient more recently, as well as in other industrial applications (i.e., bioenergy). The capability of seaweed to contribute wellbeing and health in livestock is mediated by bioactive compounds that are synthesized by a few seaweed species (8–11). Some of these bioactive compounds [e.g., bromoform in red species and polyphenols or phlorotannins in brown species (12–14)] are associated with a reduction in methanogenesis in the rumen. Many seaweed species have been evaluated through *in vitro* studies, and some have been shown to reduce CH_4_ production in the rumen (13, 15–17). For example, in an *in vitro* study, red seaweeds (*Gracilaria vermiculophyla* and *Gigartina* sp.) reduced CH_4_ production [mL/g dry matter (DM)] by 39 and 36%, respectively, when supplemented with meadow hay (Holstein cow) at a 25% of DM basis (16). When brown seaweeds (*Ulva* sp. and *Sargassum horneri*) were supplemented at 4% of DM to a total mixed ration (TMR), *in vitro* CH_4_ production (mL) was reduced by 5.75 and 3.69%, respectively [donor animals: Holstein cows; (17)]. In another *in vitro* study, extracts of five different red seaweed species (*Grateloupia lanceolata, Hypnea japonica, Pterocladia capillacea, Chondria crassicaulis*, and *Gelidium amansii*) reduced the CH_4_ production (mL/g DM) by 11.9–50.6%, when supplemented at 0.25 mg/mL (DM basis) to timothy hay [donor animals: Hanwoo cows; (18)]. The reduction in CH_4_ was at least in part caused by a reduction in methanogen abundance. Extracts of brown seaweeds (*Undaria pinnatifida, Sargassum fusiforme*, and *Sargassum fulvellum*) also reduced *in vitro* CH_4_ production (mL/g DM) by 21.3–26.8% when supplemented at 0.25 mg/mL (DM basis) to a mixture of timothy hay and corn grain [donor animals: Hanwoo cow; (13)]. Furthermore, red seaweed (*Asparagopsis taxiformis*; ATAX) has been shown to reduce CH_4_ production [mL/g organic matter (OM)] by 95–99% when supplemented at 5% OM to a TMR fed to Holstein and Jersey cows (11) or when supplemented 2% OM to Brahman steer fed Rhodes grass (19). Recently, it has been reported that *Asparagopsis* spp. reduced CH_4_ production in lactating Holstein cows (19) and Angus-Hereford cross beef steers (14) when supplemented to a TMR at 0.5% OM.

The present study investigated five red seaweed species (*Amphiroa* anceps, AANC; ATAX; *Chondracanthus tenellus*, CTEN; *Grateloupia elliptica*, GELL; and *Gracilaria parvispora*, GPAR) found in Korea. These species are edible red seaweeds commonly used as food ingredients and for diverse industrial applications (production of agar, bioethanol, and textiles) (20, 21). Many studies have confirmed that these species produce certain secondary metabolites that have positive effects on human and animal health. For example, CTEN and GPAR have radical-scavenging activities (22, 23). Kim et al. (24) and Lee et al. (25) reported that the bromophenols contained in GELL have antioxidant, anticancer, and anti-inflammatory effects. AANC contains many secondary metabolites, including tannins, which can have antimicrobial and pharmacological activities (26). Based on the bioactivities of various compounds from the extracts of these five red seaweeds, we hypothesized that they may affect the microbiota, leading to a shift in the fermentation pattern and reduction in CH_4_ production. To date, the effects of red seaweeds on microbiota and their functions have been rarely investigated, and their effects on fermentation and CH_4_ production are still unclear, except for the effect of ATAX (11, 19) and GPAR (16). Therefore, the main objectives of this study were to (1) identify total flavonoids and polyphenols in the extracts of these five red seaweeds; (2) examine the effects of supplementation with their extracts on fermentation characteristics and CH_4_ production; and (3) investigate the effects of supplementation with their extracts on microbial composition and functions.

## Materials and methods

### Ethics statement

All experimental protocols were approved by the Animal Care and Use Committee (Approval ID: GNU-180130-A0007) of the Gyeongsang National University (Jinju, Gyeongsangnam-do, Korea). All experimental procedures were performed according to the guidelines and regulations set out by this governing body.

### Red seaweed extract preparation

Five different red seaweed extracts, AANC, ATAX, CTEN, GELL, and GPAR, were provided by the Marine Biodiversity Institute of Korea (MABIK, Seocheon, Korea). The specific information on red seaweed extracts is indicated in Supplementary Table 1. Each fresh seaweed was cut or crushed into small pieces, freeze-dried, and ground into a fine powder. Then, 50 g/L powder was extracted with 70% ethyl alcohol, using an ultrasonic cleaner (WUC-N30H; Daihan Scientific, CO., Ltd, Seoul, Korea). Subsequently, stock solution (50 mg/mL) of each extract was dissolved in dimethyl sulfoxide (Sigma-Aldrich Chemical Co., St. Louis, MO, USA) and diluted using culture media before *in vitro* incubation.

### Chemical analysis

The chemical composition of the incubated substrates is shown in Table 1. Prior to chemical analysis, timothy hay and corn grain were dried at 65°C for 48 h to measure DM. Dried samples were ground with a Wiley mill (Arthur Thomas Co., Philadelphia, PA) fitted with a 1 mm screen. Timothy hay and corn grain samples were analyzed for DM, crude protein, ether extract, and ash contents, which were determined as described by AOAC (1990; method 934.01, 954.01, 920.39, and 942.05, respectively) (27). The content of neutral detergent fiber (aNDF), acid detergent fiber, and lignin was analyzed according to Van Soest et al. (28). The aNDF content was analyzed using heat-stable α-amylase and expressed inclusive of residual ash. Non-fiber carbohydrate content was estimated based on the following equation: non-fiber carbohydrate = 100–(crude protein + ether extract + ash + aNDF). Detailed analysis for total flavonoid and polyphenol was performed as described by Choi et al. (13). Total flavonoid and polyphenol concentration was measured using a microplate reader (SpectraMax M5, Molecular Devices, Sunnyvale, CA, USA) at 510 and 750 nm, respectively.

**Table 1:** Chemical composition of substrates used in the *in vitro* experiment.

**Item**	**Timothy hay**	**Corn grain**
**Chemical composition (% of DM)**		
Dry matter	92.1	85.4
Organic matter	95.6	98.8
Crude protein	8.40	7.65
Neutral detergent fiber	65.1	23.7
Acid detergent fiber	34.8	10.7
Ether extract	1.57	3.30
Non-fiber carbohydrate	20.5	64.2
Calcium	0.35	0.02
Phosphorus	0.23	0.22

All values represent the mean of triplicate analysis. DM, dry matter. NDF assayed with heat-stable amylase and expressed inclusive of residual ash.

### Experiment procedures

Two cannulated non-lactating Hanwoo cows [average body weight (BW) 506 kg] were used as rumen fluid donors. The cows were fed a standard diet composed of 600 g/kg timothy hay and 400 g/kg of commercial concentrate mix at 2% DM of their BW. The cows had free access to water. Rumen fluid samples were collected from multiple rumen sites (dorsal, ventral, cranial, and caudal) 2 h before morning feeding. The rumen content was directly collected by hand through the rumen cannula. To collect the rumen fluid, the contents were strained through four layers of cheesecloth into a pre-warmed Duran bottle (1 L) leaving no headspace. Equal volumes of the freshly strained rumen fluid from two cows were combined. In the laboratory, the inoculum was kept in a water bath at 39°C. The initial pH of the inoculum was between 6.86 and 6.91. The *in vitro* buffer (artificial saliva) was prepared as described by McDougall (29) and mixed with rumen fluid in a ratio of 1:2 (v/v), under strictly anaerobic conditions. In the bottle, 0.5 g of grounded substrates (0.3 g of timothy hay and 0.2 g of corn grain, DM basis) was placed into a nylon bag (pore size 50 ± 10 μm, R510, Ankom Technology, NY, USA). Treatments were as follows: substrate mixture without seaweed extract (CON), substrate mixture supplemented with seaweed extracts (0.25 mg/mL), and blank (without substrate mixture). The dosage of seaweed extract was chosen based on a previous study (13). The mixture of rumen fluid and *in vitro* buffer (40 mL/bottle) was accurately dispensed into each bottle under a stream of CO_2_. The bottles were capped with a butyl rubber stopper and placed in a shaking incubator (120 rpm) at 39°C for 6, 12, 24, 36, and 48 h. Three incubation runs were conducted on three separate days. Triplicate samples of each treatment were incubated in each run and time point. Three blank bottles with no substrate were used to correct for gas production arising from the inoculum.

### Sampling and measurements

At each time point, the gas production was measured using a manual pressure transducer (Laurel Electronics, Inc., Costa Mesa, CA, USA). The readings of the pressure transducer were converted to gas volume (mL) using an equation developed for our laboratory conditions (30).


$$\begin{array}{l} \text{V}=\left(\text{P}-11.015\right)/8.5502\left(\text{n}=144,{\text{R}}^{2}=\;0.999\right) \end{array}$$

where V = gas volume (mL) and P = measured pressure (psi).

Headspace gas samples (6 mL) were taken from each bottle with a 10 mL gas-tight syringe and stored in a vacuum test tube (Vacutainer, Becton Dickinson, Franklin Laker, NJ, USA). The concentration of CH_4_ in the gas samples was assayed by gas chromatography (Shimadzu, GC-2010 PLUS, Japan) equipped with HP-PLOT Q capillary column (I.D. 0.53 mm, L.30 m). A flame ionization detector (FID) with a methanizer was used to analyze CH_4_ concentration. Column, injector, and detector temperatures were controlled with 35, 200, and 250°C, respectively, and helium and hydrogen (H_2_) gases were used as carrier and combustion gases, respectively. The total production of CH_4_ was calculated according to López et al. (31) as follows:


$$\begin{array}{l} {\text{CH}}_{\text{4}},\text{mL}={\text{CH}}_{\text{4}}\text{concentration }(\text{mL/mL})\times \;(\text{Total gas},\text{ mL}\\ \;+\text{ Headspace},\;80\text{ mL}) \end{array}$$

At each time point, the pH was determined using a pH meter (S220, Mettler-Toledo, Greifensee, Switzerland). The sample of liquid cultures (5 mL from each vial) was collected to analyze volatile fatty acid (VFA) and ammonia nitrogen (NH_3_-N). Quantification of VFA was done using high-performance liquid chromatography (L-2200, Hitachi, Tokyo, Japan) according to Adesogan et al. (32). Ammonia nitrogen was quantified as described by Chaney and Marbach (33) using a spectrometer (Model 680, Bio-Rad Laboratories, Hercules, CA, USA) at 630 nm absorbance. Fluid sub-samples (1.8 mL) collected after 24 h of incubation were stored at −80°C until DNA extraction.

### *In vitro* dry matter degradability

The nylon bags were washed with running tap water until the water became clear. Subsequently, they were dried in the oven at 105°C for 24 h to determine the apparent *in vitro* DM degradability (IVDMD). *In vitro* DM degradability was calculated as the equation below, where DM_I_ means initial DM and DM_F_ means finished DM.


$$\begin{array}{l} \text{IVDMD }\left(\text{\%}\right)=\;\frac{weight\;of\;DM_{I}-weight\;of\;DM_{f}}{weight\;of\;DM_{I}}\times 100 \end{array}$$

### DNA extraction, PCR amplification, and sequencing

The genomic DNA was extracted using repeated bead beating and column extraction followed by extraction using a QIAamp Fast DNA Stool Mini Kit (Qiagen, Hilden, Germany) as described by Yu and Morrison (34). The quality and quantity of extracted DNA were analyzed using a NanoDrop ND-2000 spectrophotometer (Thermo Fisher Scientific Inc., Wilmington, DE, USA). Quantitative real-time PCR was done using a CFX 96 Touch system (Bio-Rad Laboratories, Inc.) by using several primer sets described in Supplementary Table 2, to quantify total bacteria, ciliate protozoa, fungi, and total methanogens following our previous study (13). The amplicon targeting the V3–V4 region of 16S rRNA genes was processed and sequenced by Macrogen (Macrogen Inc., Seoul, Korea). In brief, indexed 16S rRNA amplicon libraries from both prokaryotes and eukaryotes were amplified using 341F (5′ CCTACGGGNGGCWGCAG-3′) and 805R (5′-GACTACHVGGGTATCTAATCC-3′) universal primers (35) with a unique barcode for each rumen DNA sample. The amplicon libraries were sequenced using the 2 × 300 paired-end protocol on the Illumina MiSeq platform (San Diego, CA, USA).

### Bioinformatics analysis

The amplicon sequencing data were analyzed using the QIIME2 platform (version 2021.02) (36). Briefly, reads were denoised, dereplicated, and filtered (Phred Q < 25) for chimeras to generate Amplicon Sequence Variants (ASVs) according to the recommended parameters in the DADA2 workflow (37). The identified sequences from this study are available in the NCBI sequence read archive (accession numbers: PRJNA830647).

Taxonomy was assigned to the ASVs using a pre-trained classifier Silva (SSU138) 16S rRNA gene database (38). Major classified taxa, which were detected in over 50% of the samples at least one of the treatments, were discussed in this study. Alpha- and beta-diversity analyses were performed with the rarefied ASV table using the lowest sequence count (21,703 ASVs). Richness (observed ASVs and Chao1 estimates), Evenness, Simpson's index, Shannon's index, and Faith's phylogenetic diversity were calculated based on the rarefied ASV table. Principal coordinate analysis (PCoA) of Bray Curtis, Jaccard, weighted UniFrac, and unweighted UniFrac distance analysis was used to investigate the dissimilarity of overall microbiota between treatments. The reconstruction and functional prediction of the metabolic pathways of the metagenome, gene families, and enzymes were performed using the PICRUSt2 (v.2.4.1) software, with the recommended scenario and settings (39). Pearson's correlation was performed to explore the relationships among prokaryotic microbiota (existing in at least 50% of the samples), fermentation, and gas parameters. We inferred co-occurrence networks using the SparCC algorithm (40) for calculating the correlation strength and significance of major prokaryotic genera (relative abundance > 0.1%) as implemented in FastSpar (41). To determine correlations, FastSpar was run with 50 iterations, including 1,000 bootstraps to infer *P*-values.

### Statistical analysis

The data obtained from *in vitro* experiment (pH, IVDMD, NH_3_-N, VFA, and gas parameters) and the absolute abundance of ciliate protozoa, fungi, and total methanogens were analyzed using the GLIMMIX procedure of SAS software (version. 9.4, SAS Institute Inc., Cary, NC, United States).


$$\begin{array}{l} Y_{ijk}=\mu +\alpha_{i}+\beta_{j}+\gamma_{k}+\;\varepsilon_{ijkl} \end{array}$$

where Y_*ijk*_ is the experimental data, *μ* is the overall mean, *α*_*i*_ is the fixed effect of dietary treatments (*i* = 1 to 6), *β*_*j*_ is the fixed effect of incubation times (*j* = 1 to 5), *γ*_*k*_ is the random effect of the fermentation trials (*k* = 1 to 3), and ε_ijkl_ is the unexplained random error. Differences between treatment means were determined by Tukey's honest significant differences (HSD) test. Statistical significance was declared at *P* < 0.05, and a trend was discussed when 0.05 < *P* < 0.10. To assess the effects of the different treatments on the changes in a microbial community, permutational multivariate analysis of variance (PERMANOVA) with 9,999 random permutations was performed using the QIIME2 software package. Multiple comparisons for the relative abundance of the classified microbial taxa (>0.1%), some of the predicted Kyoto encyclopedia of genes and genomes (KEGG) pathways, and modules (>0.1%) were statistically analyzed by the Tukey HSD test. To identify the difference in microbial populations in five different dietary treatments to characterize the microbial population at the genera level using linear discriminant analysis (LDA) effect size (LEfSe) implemented in the Galaxy web application (42). We also identified functions of microbiota based on KEGG pathways and modules using LEfSe analysis. The normalized ASV counts in each sample were used as the input for the LEfSe analysis. Differences among classes were determined using the non-parametric Kruskal Wallis test, at a significance level of *P* < 0.05 and with a threshold LDA score of 2.0. The LEfSe was analyzed using the Wilcoxon rank sum test, with *P* < 0.05 again taken to indicate significance. Pearson's correlation coefficients among prokaryotic microbiota, fermentation, and gas parameters were calculated using the PROC CORR procedure of SAS and visualized using R package corrplot (v. 4.0.2). Only significant correlation coefficients (|*r*| > 0.7, *P* < 0.05) were determined for generating the correlation and network analysis.

## Results

### Total flavonoid and polyphenol profiles of red seaweed extracts

**Figure 1:**
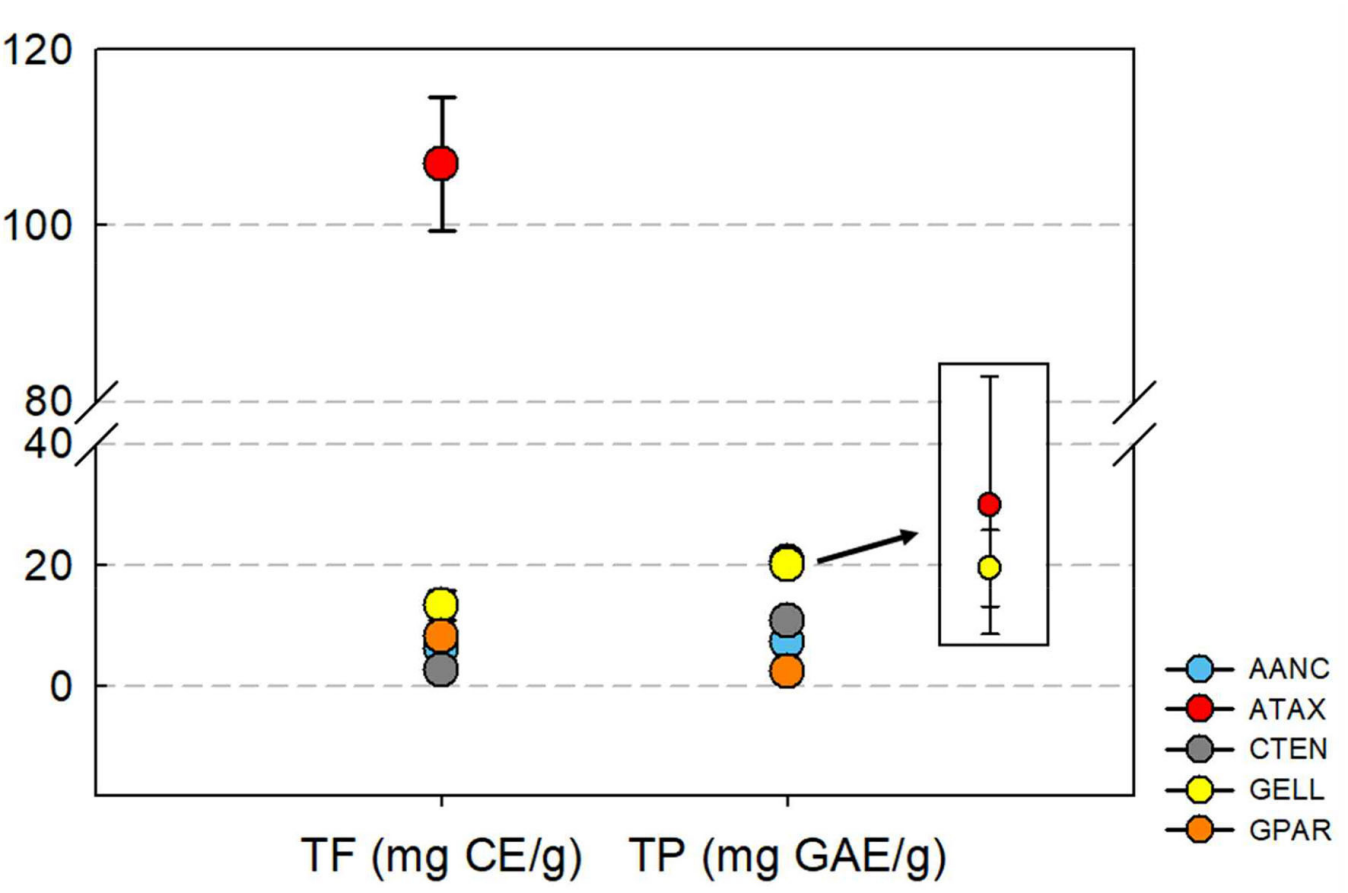
Concentration of total flavonoid and polyphenol in red seaweed extracts. TF, total flavonoid; CE, Catechin equivalent; TP, total polyphenol; GAE, garlic acid equivalent; AANC, *Amphiroa anceps*; ATAX, *Asparagopsis taxiformis*; CTEN, *Chondracanthus tenellus*; GELL, *Grateloupia elliptica*; GPAR, *Gracilaria parvispora*. Bars represent standard error of triplicate analysis.

The total flavonoid and polyphenol contents are shown in Figure 1. Total flavonoid content varied between species and was highest for ATAX [106.9 ± 7.59 mg catechin equivalent (CE)/g] followed by GELL (13.4 ± 2.34 mg CE/g), GPAR (8.20 ± 0.56 mg CE/g), AANC (6.24 ± 1.91 mg CE/g), and CTEN (6.24 ± 1.91 mg CE/g). The total polyphenol content was highest in ATAX [20.6 ± 1.01 mg garlic acid equivalent (GAE)/g] followed by GELL (20.1 ± 0.30 mg GAE/g), CTEN (10.8 ± 0.25 mg GAE/g), AANC (7.43 ± 0.42 mg GAE/g), and GPAR (2.36 ± 0.19 mg GAE/g). The ATAX extract was higher in total flavonoid and polyphenol content than the other seaweed extracts.

### *In vitro* fermentation characteristics

**Figure 2:**
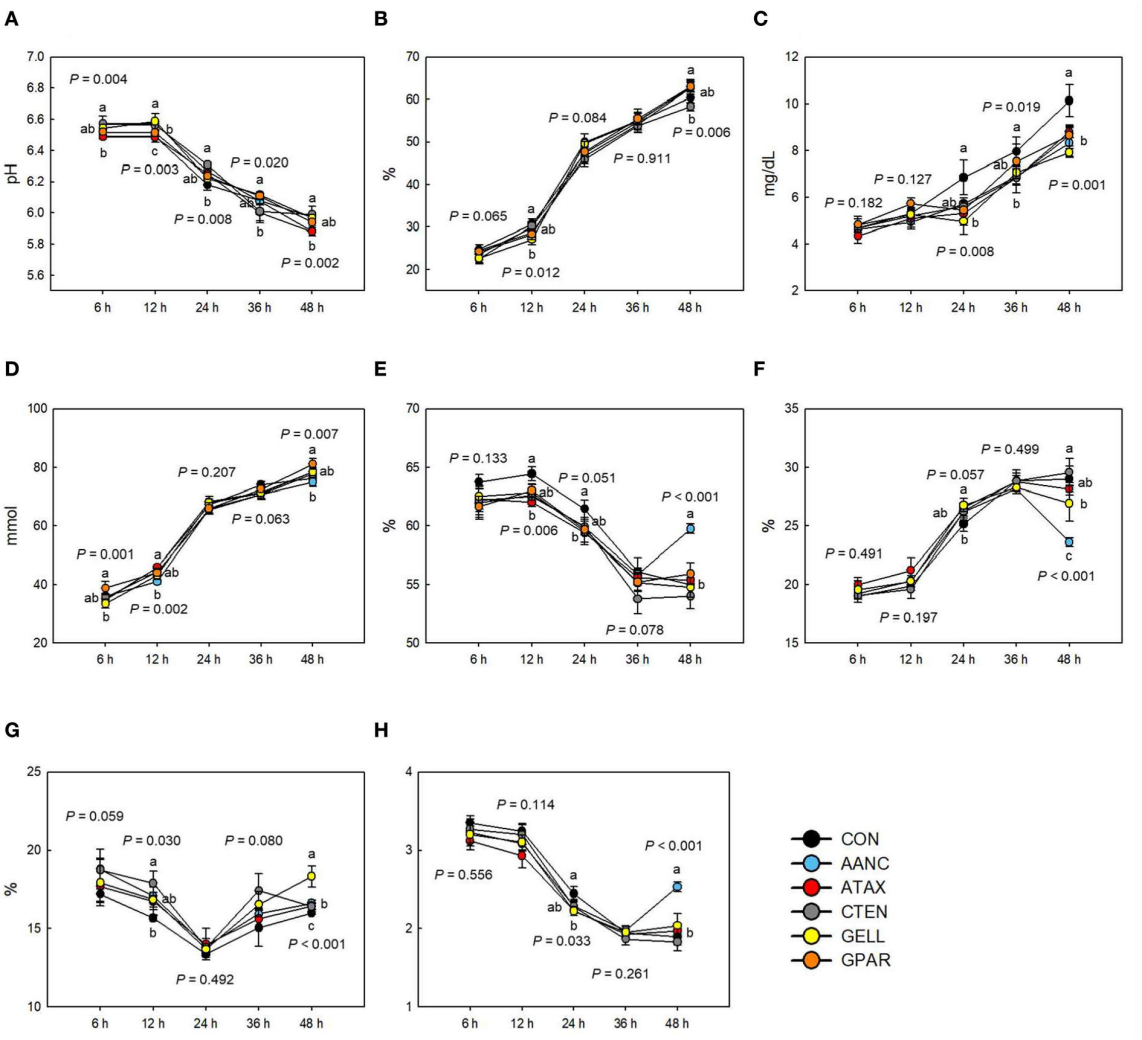
Effects of red seaweed extracts on *in vitro* fermentation characteristics. IVDMD, *in vitro* dry matter degradability; NH_3_-N, ammonia nitrogen; VFA, volatile fatty acid; AP, acetate to propionate; CON (black circle), without seaweed extracts; AANC (sky blue circle), *Amphiroa anceps*; ATAX (red circle), *Asparagopsis taxiformis*; CTEN (dark gray circle), *Chondracanthus tenellus*; GELL (yellow circle), *Grateloupia elliptica*; GPAR (orange circle), *Gracilaria parvispora*. Data were analyzed using seaweed extracts dose amount: 0.25 mg/mL, based on a 5% basis of substrate mixture. Bars represent the standard error of triplicate analysis. *P*-values represent multiple comparison output of each incubation time. ^a−*c*^Means with different superscript letters indicate a significant difference (*P* < 0.05). **(A)** pH, **(B)** IVDMD (%), **(C)** NH_3_-N (mg/dL), **(D)** Total VFA (mmol), **(E)** Acetate (%), **(F)** Propionate (%), **(G)** Butyrate (%), and **(H)** AP ratio.

The effects of the red seaweed extract on *in vitro* fermentation characteristics are shown in Figure 2. The pH was affected (*P* < 0.05) by red seaweed extracts, but to different magnitudes, depending on the sampling time point. After 6, 12, and 24 h of incubation, pH in response to AANC, CTEN, and GELL was higher (*P* < 0.01) compared to the CON. However, after 48 h of incubation, pH in response to AANC and ATAX was lower than (*P* < 0.01) that of the CON, whereas no significant differences were observed in GELL. The IVDMD after 6 and 24 h in all incubations containing seaweed extracts tended to be lower (6 h: *P* = 0.065 and 24 h: *P* = 0.084) compared to the CON. After 12 h of incubation, IVDMD in response to GELL was lower compared to the CON (*P* = 0.012). Ammonia nitrogen concentrations after 24, 36, and 48 h in all incubations containing seaweed extracts were lower compared to the CON (*P* < 0.05). After 6 and 48 h of incubation, total VFA concentration in response to AANC was lower (*P* < 0.01) compared to the CON. After 12 and 24 h of incubation, the molar proportion of acetate in response to ATAX was lower (*P* < 0.05) compared to the CON. However, after 48 h of incubation, the molar proportion of acetate in response to AANC was higher (*P* < 0.001) than that of the CON. After 24 h of incubation, the molar proportion of propionate in all incubations containing seaweed extracts tended to be higher (*P* = 0.057) compared to the CON. After 48 h of incubation, the molar proportion of propionate in response to AANC was lower (*P* < 0.001) than that of the CON. After 12 and 48 h of incubation, the molar proportion of butyrate in response to CTEN (12 h: *P* = 0.030) and GELL (48 h: *P* < 0.001) was higher than that of the CON. After 24 h of incubation, the acetate to propionate (AP) ratio in response to ATAX and GELL was lower (*P* = 0.033) compared to the CON. After 48 h of incubation, the AP ratio in response to AANC was higher (*P* < 0.001) than that of the CON.

### Total gas and CH_4_ production

**Figure 3:**
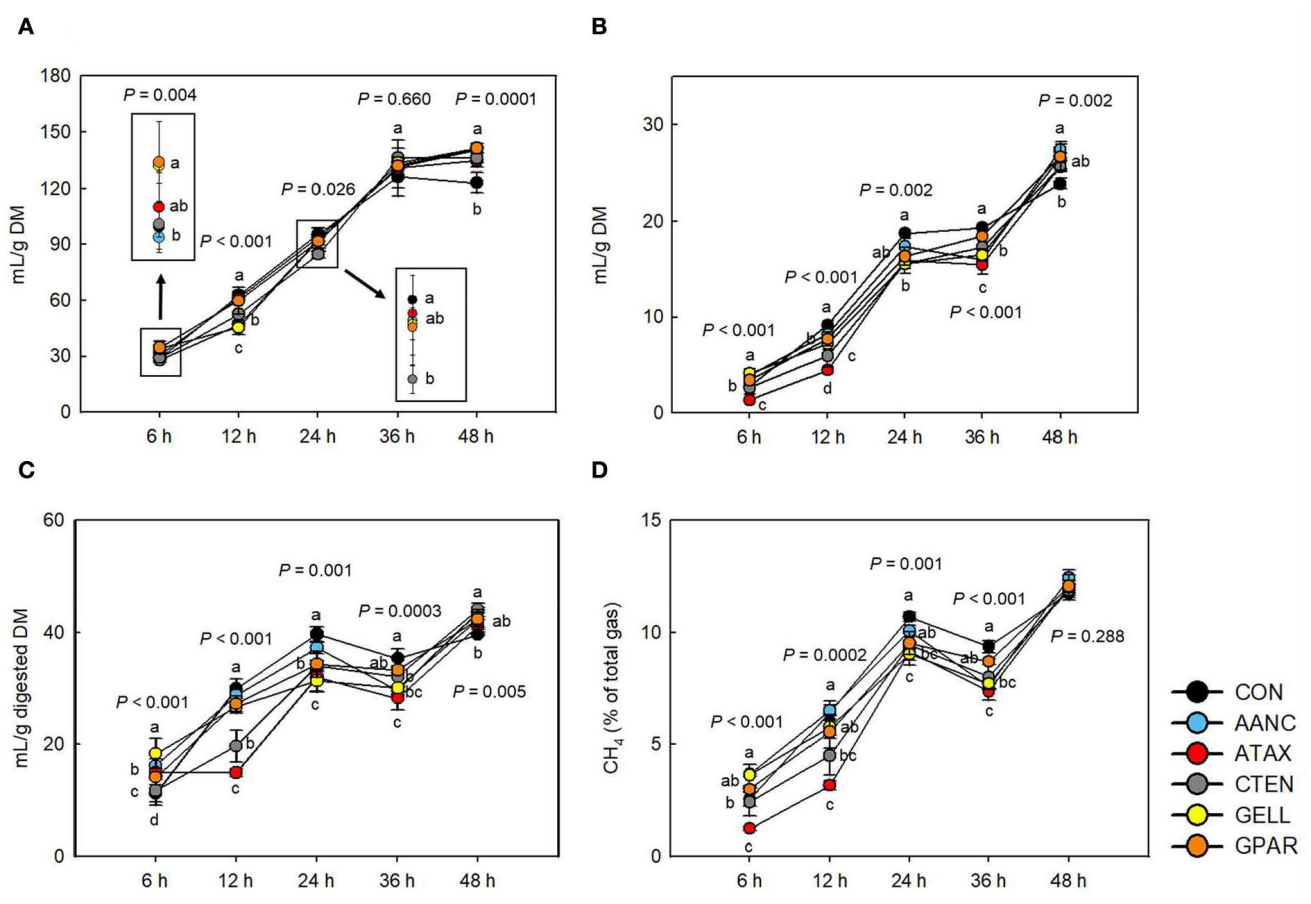
Effects of red seaweed extracts on *in vitro* total gas and methane production. CH_4_, methane; DM, dry matter; CON (black circle), without seaweed extracts; AANC (sky blue circle), *Amphiroa anceps*; ATAX (red circle), *Asparagopsis taxiformis*; CTEN (dark gray circle), *Chondracanthus tenellus*; GELL (yellow circle), *Grateloupia elliptica*; GPAR (orange circle), *Gracilaria parvispora*. Data were analyzed using seaweed extracts dose amounts: 0.25 mg/mL, 0.25 mg/mL, based on a 5% basis of substrate mixture. Bars represent the standard error of triplicate analysis. *P*-values represent multiple comparison output of each incubation time. ^a−*c*^Means (*n* = 3) with different superscript letters indicate a significant difference (*P* < 0.05). **(A)** Total gas (mL/g DM), **(B)** CH_4_ (mL/g DM), **(C)** CH_4_ (digested DM), and **(D)** CH_4_ (% of total gas).

As shown in Figure 3, after 12 h of incubation, supplementation with AANC and GELL resulted in the lowest total gas production (mL/g DM, *P* < 0.001) compare to the CON. After 48 h of incubation, total gas production (mL/g DM) in all red seaweed extracts was higher (*P* < 0.001) compared to the CON. Methane production (mL/g DM) largely followed a similar pattern to that of total gas production (mL/g DM) until 24 h of incubation. Differences were foremost observed after 36 h of incubation. Compared to the CON, the highest CH_4_ reduction (mL/g DM, mL/g digested DM, and % of total gas production) was observed in ATAX (51.3, 50.1, and 51.5%, respectively, *P* < 0.001) at all the time points, except for 48 h after the onset of incubation. After 6 h of incubation, AANC, GELL, and GPAR led to higher CH_4_ production (mL/g DM), mL/g digested DM, and % of total gas production (23.4–49.4, 25.8–63.4, and 17.6–43.7%, respectively, *P* < 0.001) than that of the CON. However, after 36 h of incubation, all red seaweed extracts except GPAR reduced CH_4_ production (mL/g DM, 4.66–19.9%, *P* < 0.001), mL/g digested DM (6.01–20.2%, *P* = 0.0003), and CH_4_ production (% of total gas, 7.14–21.5%, *P* < 0.001) that of the CON.

### Microbial composition

**Figure 4:**
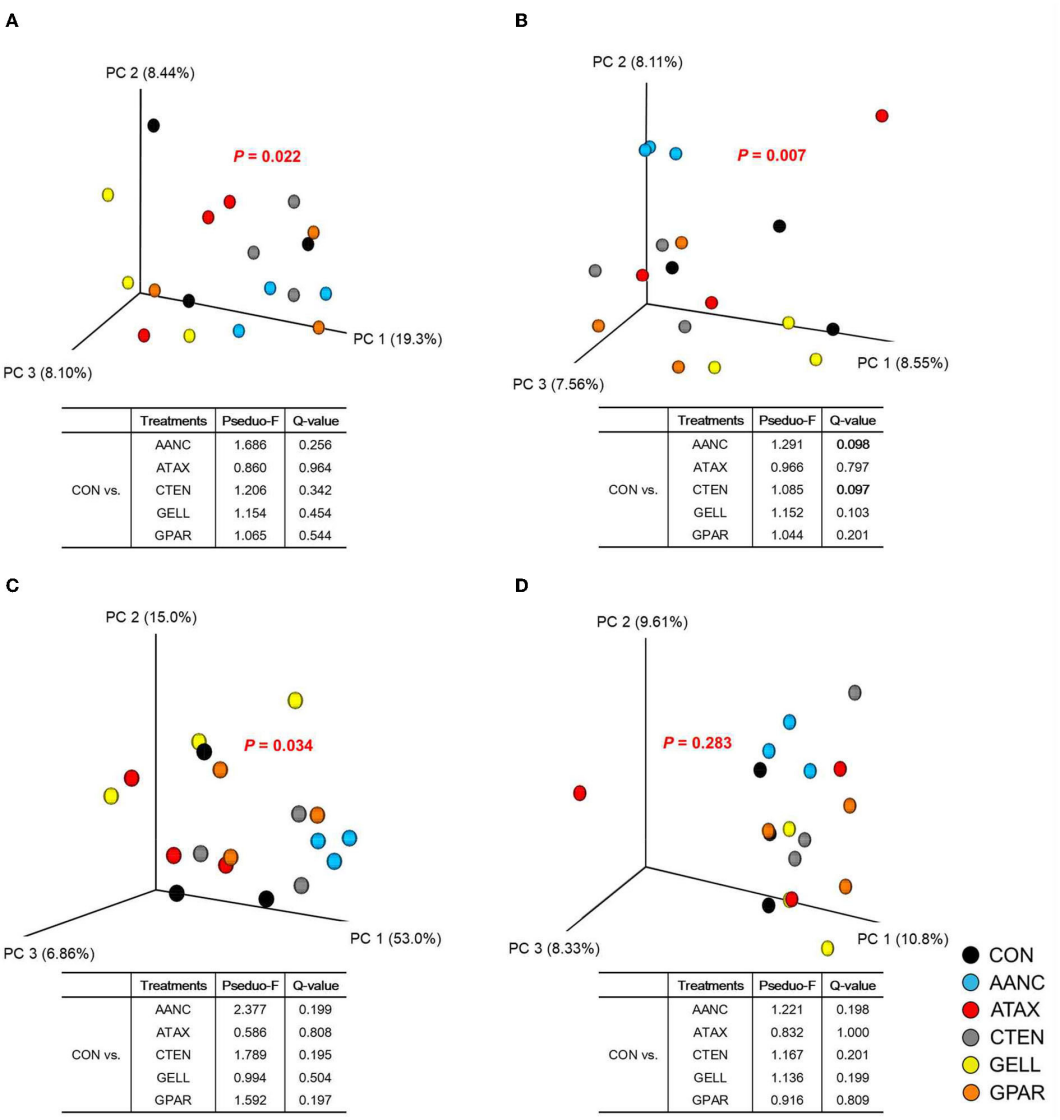
Principal coordinates analysis based on the **(A)** Bray-Curtis, **(B)** Jaccard, **(C)** Weighted Unifrac, and **(D)** Unweighted Unifrac distances of the red seaweed extracts in the *in vitro* cultures. PERMANOVA results of the pairwise comparison of the overall microbiota structures between the control and each red seaweed treatment were included (after 24 h of *in vitro* incubation). CON (black circle), without seaweed extracts; AANC (sky blue circle), *Amphiroa anceps*; ATAX (red circle), *Asparagopsis taxiformis*; CTEN (dark gray circle), *Chondracanthus tenellus*; GELL (yellow circle), *Grateloupia elliptica*; GPAR (orange circle), *Gracilaria parvispora*.

A total of 1,035,111 16S rRNA sequences were generated by the 16S rRNA sequence analysis. After the removal of low-quality, non-targeted and chimeric amplicons by QIIME2 (Q score > 25), 516,939 sequences (50% of the raw reads) were obtained with an average of 28,719 ± 2,824 sequences per sample. As shown in Table 2, alpha-diversity measurements of the prokaryotic community (observed ASV, Chao 1 estimates, evenness, Shannon's index, and faith's PD) did not differ between CON and red seaweed extracts group. However, Simpson's index was higher in CTEN than that of the CON. Regards to beta-diversity compared by PERMANOVA was shown in Figure 4. The Bray-Curtis, weighted Unifrac, and unweighted Unifrac distances of the each of red seaweed extracts did not differ from CON. However, Jaccard distance tended to be affected by supplementation AANC and CTEN (*Q* = 0.098 and 0.097) than that of the CON. In the overall comparison, it was differed from CON in Bray-Curtis (*P* = 0.022), Jaccard (*P* = 0.007), and weighted Unifrac distance (*P* = 0.034), whereas no differences were observed in unweighted Unifrac distance.

**Table 2:** Alpha diversity measurements of the microbiota 24 h of *in vitro* incubation.

**Measurement**	**CON**	**Treatments**					**SEM**	***P*** **-value**
		**AANC**	**ATAX**	**CTEN**	**GELL**	**GPAR**		
**Diversity**							
Observed ASVs	798	742	745	840	831	799	51.4	0.325
Chao1 estimates	801	744	747	843	832	800	51.8	0.330
Evenness	0.90	0.90	0.90	0.90	0.90	0.89	0.00	0.274
Simpson's index	0.99	0.99	0.99	1.00	1.00	0.99	0.00	0.043
Shannon's index	8.66	8.53	8.59	8.76	8.74	8.62	0.09	0.169
Faith's PD	47.1	46.0	46.3	48.9	49.1	47.0	2.14	0.603

All values represent the mean (n = 3, n means separate run). SEM, standard error of the mean; amplicon sequence variant; PD, phylogenetic diversity; CON, without seaweed extracts; AANC, *Amphiroa anceps*; ATAX, *Asparagopsis taxiformis*; CTEN, *Chondracanthus tenellus*; GELL, *Grateloupia elliptica*; GPAR, *Gracilaria parvispora*.

The Venn diagram showed shared prokaryotic genera between CON and red seaweed extracts (Supplementary Figure 1). Over 110 genera were shared by each of the red seaweed extracts and CON. The most shared red seaweed extract was GPAR which shared a total of 124 genera.

The major taxa with relative abundance above 0.1% in at least one group are shown in Supplementary Figure 2. At the phylum level, nine phyla had a relative abundance > 0.1%, and Bacteroidota, Firmicutes, Proteobacteria, and Fibrobacterota were the four dominant phyla. At the genus level, three dominant genera were *Prevotella*, Rikenellaceae RC9 gut group, and F082, while the genus *Denitrobacterium* only found in red seaweed extracts supplementation. The differentially abundant family and genus levels between the CON and red seaweed extracts group were identified using LEfSe (Table 4). At the family level, red seaweed extracts supplementation was more enriched in the relative abundance of three families within Acholeplasmataceae (GELL), WCHB1-41 (GPAR), and Eggerthellaceae (ATAX), whereas there were no families enriched in the CON, AANC, and CTEN. At the genus level, a total of six genera including UG Ruminococcaceae (ATAX), *Denitrobacterium* (ATAX), *Candidatus Saccharimonas* (CTEN), *Anaeroplasma* (GELL), UG Succinivibrionaceae (GELL), and *Muribaculaceae* (GPAR) were found to be enriched in the red seaweed extracts supplementation, whereas there were no genera enriched in the CON and AANC.

The RT-PCR results showed that the abundance of total bacteria, ciliate protozoa, and fungi had no significant differences observed between CON and red seaweed extract supplementation (Table 4). However, the abundance of total methanogens was lower (*P* = 0.016) in AANC and GPAR than that of the CON.

### Predicted functions of the microbiota

No significant difference in the predicted functional features of the microbiota against seven different databases (i.e., COG, PFAM, EC, MetaCyc Pathways, KEGG orthologs, KEGG modules, and KEGG pathways) was detected between the CON and red seaweed extract (Supplementary Table 3). The overall predicted functional features are shown in Table 5. According to LEfSe analysis, two KEGG pathways were enriched in AANC supplementation (taurine and hypotaurine metabolism, ko00430; and glyoxylate and dicarboxylate metabolism, ko00630). In the KEGG modules, four predicted functional features were found to be enriched in the GELL supplementation (threonine biosynthesis, M00018; guanine ribonucleotide biosynthesis; M00050; NAD biosynthesis, M00115; and coenzyme A biosynthesis, M00120), whereas C5 isoprenoid biosynthesis (M00096) was enriched in CON. Although not identified as a biomarker by the LEfSe analysis, we found some significant KEGG pathways or modules related to fermentation characteristics (ko00280, valine, leucine and isoleucine degradation; ko00071, fatty acid degradation; and M00149, succinate dehydrogenase) and CH_4_ production (ko00680, CH_4_ metabolism; M00176, assimilatory sulfate reduction; and M00344, Formaldehyde assimilation belong to CH_4_ metabolism).

### Correlation analysis

**Figure 5:**
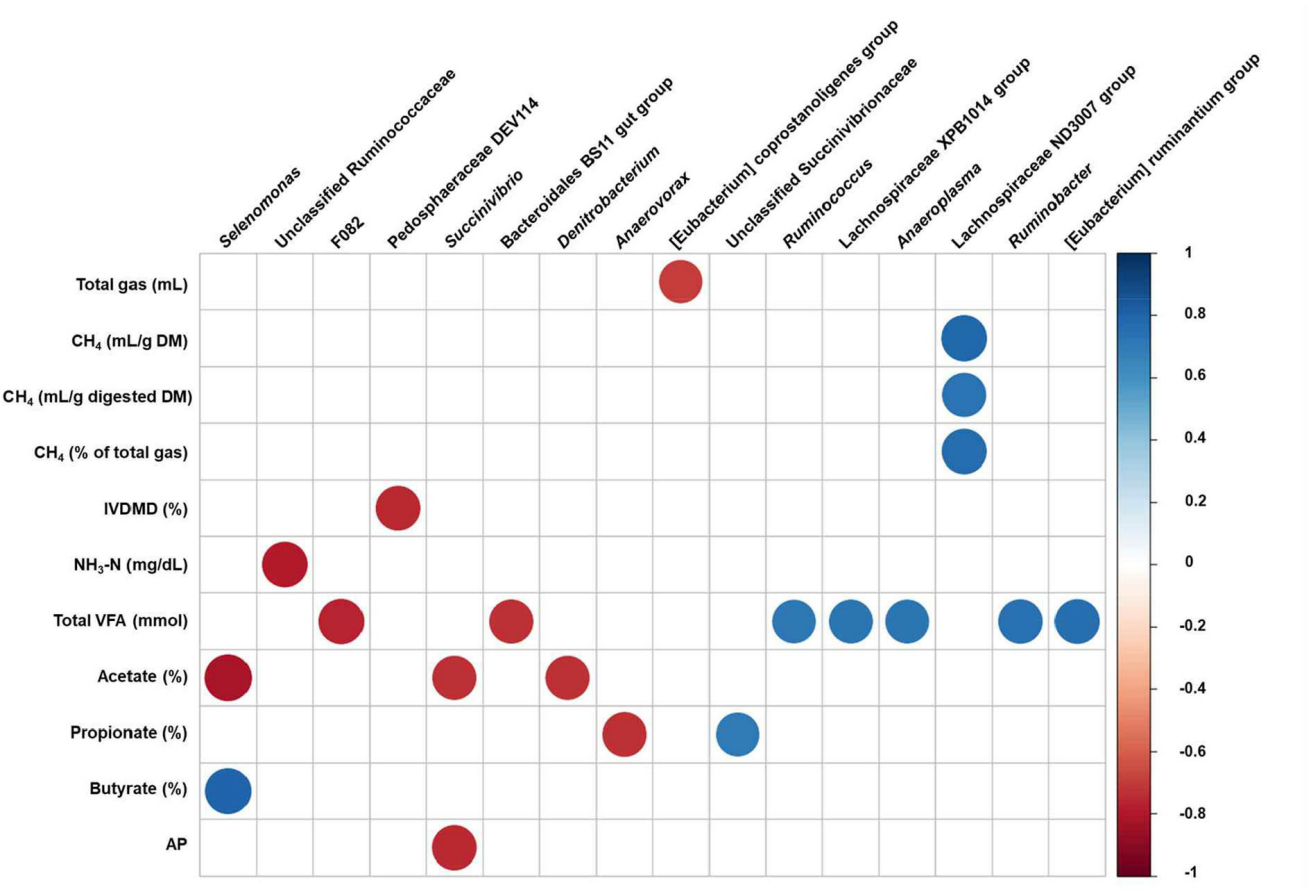
Pearson's correlation coefficients between the relative abundance of differentially abundant bacterial genera and fermentation and gas parameters affected by the red seaweed extract supplementation after 24 h of *in vitro* incubation. Each row in the graph represents fermentation and gas parameters, each column represents a prokaryotic genera. Only strong significant correlation coefficients (|*r*| > 0.7, *P* < 0.05) are shown on the plot. CH_4_, methane; DM, dry matter; NH_3_-N, ammonia nitrogen; VFA, volatile fatty acid; AP, acetate to propionate; UCG, uncultured genus-level group.

To identify the associations between the fermentation and gas parameters and differently abundant taxa, we conducted a correlation analysis by calculating Pearson's correlation coefficients (Figure 5). Strong correlations (Pearson correlation coefficients, |*r*| > 0.7, *P* < 0.05) were detected by supplementation with red seaweed extracts. Total gas production was negatively correlated with [Eubacterium] coprostanoligenes group, whereas CH_4_ production (including CH_4_ digested DM and % of total gas production) was positively correlated with the Lachnospiraceae ND3007 group. The concentration of NH_3_-N and IVDMD was negatively correlated with unclassified Ruminococcaceae and Pedosphaeraceae DEV114, respectively. [Eubacterium] ruminantium group, Lachnospiraceae XPB1014 group, *Ruminobacter, Ruminococcus*, and *Anaeroplasma* were positively correlated with total VFA production, whereas Bacteroidales BS11 gut group and F082 were negatively correlated. The molar proportion of acetate was negatively correlated with *Selenomonas, Succinivibrio*, and *Denitrobacterium*, whereas molar proportion of butyrate was positively correlated with *Selenomonas*. The molar proportion of propionate was positively correlated with unclassified Succinivibrionaceae and negatively correlated with *Anaerovorax*, whereas AP was negatively correlated with *Succinivibrio*.

To infer an underlying relationship between prokaryotic microbiota by red seaweed extract supplementation, we plotted the co-occurrence network interaction. The co-occurrence network consisted of 38 nodes and 72 edges (Supplementary Figure 3), including 48 positive correlations and 24 negative correlations (|*r*| > 0.7, *P* < 0.05). Based on the centrality parameters (including degree, authority, and eigenvector centrality), F082 was the best centrality genus in red seaweed extract supplementation.

## Discussion

Flavonoids and polyphenols in seaweeds exert antimicrobial effects (43) and can interact either positively or negatively with the rumen microbiota (44). For example, flavan-3-ol, (+)-catechin (a flavonoid sub-group) can function as an alternative H_2_ sink to CH_4_ precursors in the rumen (45). In the present study, we found that ATAX was the most effective species to reduce CH_4_ production. Interestingly, ATAX had the highest total polyphenol and flavonoid content (Figure 1). Several studies reported that flavonoids and polyphenols can alter rumen fermentation and microbiota (13, 46–48). For example, Ma et al. (47) reported that supplementation with natural polyphenol resveratrol reduced the total VFA content regardless of the diet type or fermentation time. Our previous study also showed that supplementation with brown seaweed extracts (with similar or lower contents of flavonoids and polyphenols than those in red seaweed extracts) decreased the abundance of proteolytic species (13). However, different types of flavonoid and polyphenol compounds are produced in different seaweed species, explaining their different effects on the rumen microbiota (13). The concentrations of flavonoids, polyphenols, and other bioactive compounds in seaweeds can vary depending on the growing seasons, geographical locations, and post-harvest processing (49, 50). For example, most studies of ATAX used ATAX harvested in Australia (11, 14, 15, 19). Thus, it is possible that the observed CH_4_ reduction efficiency of 8–90% in the present study occurred because the content of the main anti-methanogenic bioactive compound in ATAX (bromoform) collected in Korea differs from that in ATAX collected in Australia. Furthermore, other substances in seaweed may contribute to differences in CH_4_ reduction, as suggested by Vijn et al. (51). The activity of bromoform can also be impacted by the extraction process or storage conditions, as suggested by Stefenoni et al. (52). It is also possible that the lower effectiveness in CH_4_ reduction was caused by differences in the substrate used and by microbial variations in the rumen fluid.

Our results clearly indicate that most red seaweed extracts significantly reduced CH_4_ production after 12 h of incubation, which was accompanied by decreased IVDMD, total and individual VFA, and total gas production (Figure 2). These results are consistent with those of previous studies in which ruminant diets were supplemented with red seaweed and red seaweed extracts (11, 15). Studies of red seaweeds showed that ATAX, *Halymenia floresii, Hypnea pannosa*, and *Laurencia filiformis* reduced CH_4_ production (mL/g OM) in an *in vitro* batch system by 26.5–98.9% and total gas production by 10.1–61.8% (15). Roque et al. (11) reported that supplementation with 5% ATAX (OM basis) significantly reduced total gas (mL/g OM, −51.8%) and CH_4_ production (mL/g OM, −95%). However, our study showed that total gas and CH_4_ production gradually recovered after 36 h of incubation, which appears to be associated with increased IVDMD and total VFA production. This finding is consistent with those of a previous *in vitro* study in which five red seaweed species (*Grateloupia lanceolata, Hypnea japonica, Pterocladia capillacea, Chondria crassicaulis*, and *Gelidium amansii*) were supplemented (18).

Several factors may explain the recovery of fermentation parameters after 24 h of incubation between red seaweed extract supplementation and the CON. First, the relative abundances of members of the order Bacteroidales (Rikenellaceae RC9 gut group and F082) were higher in AANC, CTEN, and GPAR than in the CON (Table 3). Pitta et al. (53) demonstrated that the Rikenellaceae RC9 gut group is a member of the Rikenellaceae family, which are fibrolytic microbes. The Rikenellaceae RC9 gut group produces acetate and propionate as fermentation end products (54) and may contribute to reducing CH_4_ production by participating in VFA production and H_2_ scavenging (55). The specific function of F082 remains unclear, but F082 taxa can ferment various substrates because of their high abundance and multifunctional characteristics, which is characteristics of these *Bacteroidetes* (56). Although F082 was found to be enriched in AANC, CTEN, and GPAR, they were negatively correlated with total VFA production in Figure 5, suggesting that F082 enriched by supplementation with red seaweed extracts does not play a crucial role in VFA production. Nonetheless, we found that both genera (Rikenellaceae RC9 gut group and F082) were important nodes in red seaweed extract based on co-occurrence network analysis (Supplementary Figure 3). Interestingly, these genera showed a co-occurrence interaction with many minor prokaryotic microbiota (i.e, Pedosphaeraceae DEV114, Papillibacter, UCG-010) whose functions are not well-understood. Thus, they may be associated with prokaryotic microbiota with diverse metabolic functions. Second, the relative abundance of *Candidatus Saccharimonas*, a potential cellulose utilizer (57), was higher following supplementation with all red seaweed extracts, suggesting that the red seaweed extracts promote the growth of *Candidatus Saccharimonas* and thus enhance its VFA production. Third, except for GPAR, the other red seaweed extracts did not affect the absolute abundance of ciliate protozoa and fungi, which play important roles in plant cell wall degradation. Taken together, red seaweed extracts may affect the growth of certain prokaryotic taxa, but the underlying mechanisms require further investigation.

**Table 3:** Major genera of bacteria (relative abundance > 0.1%) in response to supplementation of red seaweed extracts after 24 h of *in vitro* incubation.

**Genus**	**Relative abundance (%)**						**SEM**	***P*** **-value**
	**CON**	**AANC**	**ATAX**	**CTEN**	**GELL**	**GPAR**		
*Prevotella*	17.9	17.3	18.7	18.4	20.0	19.4	0.72	0.184
Rikenellaceae RC9 gut group	11.8	13.1	11.5	13.2	11.4	12.9	0.56	0.051
F082	10.5	12.3	10.2	11.4	10.2	11.1	0.49	0.036
Bacteroidales RF16 group	7.96	9.10	7.25	7.96	7.14	7.61	0.49	0.120
*Ruminobacter*	4.53	4.43	4.96	3.00	6.33	3.38	0.83	0.089
Christensenellaceae R-7 group	4.40	3.66	4.50	4.22	3.64	3.96	0.27	0.180
*Fibrobacter*	2.27	1.55	1.93	1.37	2.45	1.45	0.32	0.162
Oscillospiraceae NK4A214 group	2.23	2.03	2.33	2.35	2.06	2.31	0.15	0.535
Bacteroidales BS11 gut group	1.90	2.07	1.70	1.90	1.72	1.89	0.09	0.142
*Succinivibrio*	1.57	0.83	1.41	0.83	1.39	0.97	0.17	0.126
UCG-010	1.44	1.60	1.39	1.50	1.34	1.54	0.09	0.377
*Anaeroplasma*	1.42^ab^	0.99^b^	1.46^ab^	1.04^b^	1.72^a^	1.06^b^	0.12	0.004
*Succiniclasticum*	1.37	1.38	1.48	1.47	1.47	1.47	0.11	0.952
*Acetitomaculum*	1.25	1.03	1.31	1.25	1.07	1.18	0.09	0.297
WCHB1-41	1.20^ab^	1.14^ab^	0.99^ab^	0.99^ab^	0.80^b^	1.34^a^	0.10	0.037
Lachnospiraceae NK3A20 group	1.05	0.92	0.89	1.06	0.70	0.92	0.13	0.436
Prevotellaceae UCG-003	0.97^ab^	0.92^ab^	0.82^b^	0.96^ab^	0.96^ab^	1.01^a^	0.06	0.073
Erysipelatoclostridiaceae UCG-004	0.89	0.98	0.87	0.84	1.02	0.85	0.08	0.581
Oscillospiraceae UCG-002	0.86	1.13	0.95	0.91	0.79	0.88	0.16	0.728
*Quinella*	0.82	0.69	0.72	0.73	0.55	0.84	0.08	0.192
*Treponema*	0.77	0.48	0.73	0.56	0.91	0.50	0.13	0.116
*Ruminococcus*	0.77	0.33	0.78	0.63	0.76	0.50	0.13	0.156
*Gastranaerophilales*	0.77	0.86	0.74	0.84	0.86	0.71	0.05	0.179
MVP-15	0.68	0.66	0.62	0.60	0.56	0.56	0.04	0.365
Clostridia vadinBB60 group	0.56	0.55	0.53	0.48	0.60	0.58	0.08	0.876
p-251-o5	0.56	0.63	0.62	0.73	0.65	0.66	0.05	0.312
[Eubacterium] coprostanoligenes group	0.54	0.58	0.64	0.72	0.57	0.64	0.07	0.509
*Muribaculaceae*	0.53	0.62	0.53	0.73	0.48	0.75	0.06	0.027
Pedosphaeraceae DEV114	0.52	0.59	0.47	0.56	0.46	0.57	0.05	0.355
UG Succinivibrionaceae	0.51^ab^	0.35^b^	0.59^ab^	0.37^b^	0.69^a^	0.43^ab^	0.06	0.011
*Candidatus Saccharimonas*	0.49^a^	0.64^ab^	0.56^bc^	0.69^a^	0.54^bc^	0.60^ab^	0.02	<0.001
*Selenomonas*	0.48	0.35	0.49	0.39	0.36	0.56	0.09	0.567
*Papillibacter*	0.43	0.58	0.44	0.46	0.43	0.49	0.04	0.156
Succinivibrionaceae UCG-002	0.31	0.30	0.41	0.38	0.55	0.35	0.07	0.169
*Methanobrevibacter*	0.40	0.27	0.38	0.38	0.31	0.27	0.03	0.050
*Denitrobacterium*	0.00^b^	0.09^ab^	0.20^a^	0.14^a^	0.15^a^	0.17^a^	0.03	0.003

All values represent the mean (n = 3, n means separate run).

a−c Means in the same row with unlike superscripts differ, P < 0.05. SEM, standard error of the mean; CON, without seaweed extracts; AANC, *Amphiroa anceps*; ATAX, *Asparagopsis taxiformis*; CTEN, *Chondracanthus tenellus*; GELL, *Grateloupia elliptica*; GPAR, *Gracilaria parvispora*; UCG, uncultured genus-level group; UG, Unclassified genus.

An increase in propionate production decreases the available H_2_ for CH_4_ formation (58). Therefore, increased propionate content in the rumen is associated with reduced CH_4_ production. In the present study, the molar proportion of propionate tended to increase following red seaweed extract supplementation after 24 h of incubation (Figure 2). These results are consistent with those of previous studies (11, 18, 19). Propionate is typically produced *via* two pathways in the rumen: the succinate (major pathway) and acrylate pathways (59). Species of the genera *Prevotella, Succinivibrio*, and *Ruminobacter* ferment carbohydrates to produce succinate (60), whereas *Succiniclasticum* and *Selenomonas* ferment succinate to produce propionate through the succinate pathway (61). In the present study, the abundance of *Ruminobacter*, UG Succinivibrionaceae, and Succinivibrionaceae UCG*-*002 was higher after in response to supplementation with ATAX and GELL (Table 3). Although the relative abundance of *Succinivibrio* and *Selenomonas* did not differ between the treatments supplemented with red seaweed extract and the CON, it was negatively correlated with the molar proportion of acetate (Figure 5). In addition, unclassified Succinivibrionaceae was positively correlated with the molar proportion of propionate. Red seaweed extract supplementation may increase the activity of *Succinivibrio* to promote increased propionate production by utilizing the H_2_ typically used for CH_4_ production and instead using it for propionate production, thereby contributing to a reduction in CH_4_. Huntington et al. (62) reported that rumen fermentation of starch increases rumen propionate production. Thus, *Anaeroplasma*, a genus involved in starch digestion (63) which was predominant in GELL supplementation, may also contribute to higher propionate production.

In the rumen, the genus *Methanobrevibacter* is responsible for producing CH_4_ and accounts for approximately 61.6–74% of the total archaeal community (64–66). A reduction in CH_4_ production in the rumen is typically accompanied by significantly lower abundances of methanogens and ciliate protozoa (67). However, conflicting observations have been reported in several studies, despite the reduction in CH_4_ production (13, 68). According to Guo et al. (68), CH_4_ formation was reduced by the application of tea saponins, which lowered the activity of the mcrA gene; these results indicate that the methanogen population has methanogenic activity and that the total number of methanogens was not affected. In addition, in our previous *in vitro* study, brown seaweed extracts inhibited protozoan populations, but different partner specificities were observed within archaeal and protozoan species (13). We predicted that methanogens living in association with ciliate protozoa would not be affected to the same extent as ciliate protozoa, whereas the remaining methanogens residing freely in the rumen would be decreased by red seaweed extract supplementation. This hypothesis was supported by a previous study showing that numerous protozoan genera and methanogen species were positively or negatively correlated with CH_4_ production (69). In addition, some studies showed that variations in CH_4_ production do not necessarily result from changes in the methanogen cell density but rather are related to the community structure of the methanogens (70, 71). Only small alterations in the either prokaryotic community or Euryarchaeota were similar between the CON and red seaweed extracts in the present study (Table 4, Figure 4). Our results highlight that large-scale changes in the rumen microbial community are not a prerequisite for altering the function of the rumen microbiome, as suggested by Roque et al. (11). We could not determine whether the rumen methanogen community and ciliate protozoa composition was altered in response to supplementation with red seaweed extracts; whether the extracts further modify the methanogen composition and CH_4_ production requires further analysis. The discrepancies between CH_4_ production and CH_4_-producing microbiota may be explained by the lack of significant differences in CH_4_ metabolism in the KEGG pathway (ko00680) or module (M00344) analysis.

**Table 4:** Abundance of bacteria families and genera in response to supplementation with red seaweed extracts evaluated with LEfSe analysis after 24 h of *in vitro* incubation.

**Taxa**	**Dominance^1^**	**Relative abundance (%)**						**SEM**	**LDA**	***P*** **-value**
		**CON**	**AANC**	**ATAX**	**CTEN**	**GELL**	**GPAR**			
**Family**										
Eggerthellaceae	ATAX	0.120	0.321	0.519	0.469	0.408	0.454	0.04	3.366	0.027
WCHB1-41	GPAR	1.204	1.141	0.989	0.988	0.796	1.342	0.10	3.447	0.050
Acholeplasmataceae	GELL	1.498	1.023	1.521	1.105	1.817	1.107	0.12	3.610	0.033
**Genus**										
UG Ruminococcaceae	ATAX	0.107	0.036	0.177	0.078	0.128	0.067	0.03	2.968	0.042
*Denitrobacterium*	ATAX	0.000	0.085	0.199	0.135	0.147	0.172	0.04	3.170	0.037
*Candidatus saccharimonas*	CTEN	0.490	0.641	0.559	0.687	0.544	0.605	0.02	3.260	0.015
*Anaeroplasma*	GELL	1.419	0.991	1.455	1.043	1.717	1.058	0.12	3.610	0.033
UG Succinivibrionaceae	GELL	0.505	0.353	0.593	0.368	0.686	0.430	0.06	3.280	0.029
*Muribaculaceae*	GPAR	0.532	0.617	0.527	0.732	0.475	0.754	0.06	3.155	0.035
**RT PCR (log**_**10**_ **rrs copies/mL)**										
Total bacteria	—	10.2	10.3	10.3	10.3	10.4	10.3	0.06	—	0.672
Ciliate protozoa	—	8.57	8.60	8.62	8.63	8.57	8.49	0.06	—	0.341
Fungi	—	6.24	6.21	6.38	6.38	6.43	6.13	0.08	—	0.113
Total methanogens	—	8.56^a^	8.09^b^	8.21^ab^	8.30^ab^	8.30^ab^	8.17^b^	0.08	—	0.016

1 Only the taxa which have over 0.1% average relative abundance in at least one of the treatments were statistically analyzed by LEfSe (P < 0.05, LDA ≥ 2). All values represent the mean (n = 3, n means separate run).

ab Means in the same row with unlike superscripts differ, P < 0.05. LEfSe, linear discriminant analysis effect size; LDA, linear discriminant analysis; SEM, standard error of the mean; CON, without seaweed extracts; AANC, *Amphiroa anceps*; ATAX, *Asparagopsis taxiformis*; CTEN, *Chondracanthus tenellus*; GELL, *Grateloupia elliptica*; GPAR, *Gracilaria parvispora;* UG, unclassified genus.

Red seaweed extract supplementation can affect the important features of feed digestion (such as carbohydrate, amino acid, and lipid metabolism), as well as many fundamental microbe characteristics (such as threonine biosynthesis, NAD biosynthesis, and coenzyme A biosynthesis) (Table 5). A comparison of CON and AANC supplementation revealed enrichment in valine, leucine, and isoleucine degradation (ko00280); these amino acids are important contributors to microbial protein synthesis. According to Xue et al. (72), the enrichment of valine, leucine, and isoleucine metabolism suggests that microbial protein synthesis is increased in the rumen to enhance feed efficiency. Additionally, fatty acid degradation (ko00071) was enriched after AANC supplementation, thus increasing the yield of acetyl-CoA, which may be further used to produce energy for cellular biosynthesis (73, 74). However, the fermentation characteristics (IVDMD and total VFA) did not differ between the CON and AANC groups, indicating that AANC supplementation can alter unspecified metabolic pathways during fermentation without detrimental effects.

**Table 5:** Abundance of KEGG pathways and modules in response to supplementation with red seaweed extracts evaluated with LEfSe analysis after 24 h of *in vitro* incubation.

**Parameters**	**Dominance^1^**	**CON**	**Treatments**					**SEM**	**LDA**	***P*** **-value**	**Description**
			**AANC**	**ATAX**	**CTEN**	**GELL**	**GPAR**				
**KEGG pathways**											
ko00430	AANC	0.727	0.743	0.730	0.727	0.737	0.723	0.00	2.884	0.035	Taurine and hypotaurine metabolism
ko00630	AANC	0.670	0.680	0.673	0.673	0.680	0.677	0.00	2.457	0.047	Glyoxylate and dicarboxylate metabolism
ko00280	–	0.580^b^	0.618^a^	0.575^b^	0.608^a^	0.577^b^	0.599^ab^	0.01	–	0.015	Valine, leucine and isoleucine degradation
ko00071	–	0.380^b^	0.395^a^	0.377^b^	0.391^ab^	0.379^b^	0.386^ab^	0.00	–	0.007	Fatty acid degradation
ko00680	–	0.439	0.436	0.444	0.436	0.438	0.433	0.00	–	0.184	Methane metabolism
**KEGG modules**											
M00018	GELL	0.783	0.780	0.787	0.780	0.787	0.780	0.00	2.531	0.049	Threonine biosynthesis
M00050	GELL	0.913	0.907	0.913	0.910	0.920	0.913	0.00	2.848	0.040	Guanine ribonucleotide biosynthesis
M00096	CON	0.937	0.920	0.937	0.930	0.937	0.930	0.00	2.740	0.020	C5 isoprenoid biosynthesis
M00115	GELL	0.847	0.840	0.850	0.840	0.850	0.840	0.00	2.542	0.024	NAD biosynthesis
M00120	GELL	0.673	0.670	0.670	0.670	0.677	0.670	0.00	2.615	0.037	Coenzyme A biosynthesis
M00149	–	0.68	0.72	0.67	0.71	0.68	0.72	0.01	–	0.061	Succinate dehydrogenase
M00176	–	0.13^ab^	0.13^ab^	0.12^b^	0.12^b^	0.13^a^	0.12^b^	0.00	–	0.006	Assimilatory sulfate reduction
M00344	–	0.58	0.58	0.57	0.58	0.57	0.58	0.00	–	0.030	Formaldehyde assimilation

1 Only the KEGG pathways and modules which have over 0.1% average relative abundance in at least one of the treatments were statistically analyzed by LEfSe (P < 0.05, LDA ≥ 2). All values represent the mean (n = 3, n means separate run).

ab Means in the same row with unlike superscripts differ, P < 0.05.

LEfSe, linear discriminant analysis effect size; LDA, linear discriminant analysis; SEM, standard error of the mean; KEGG, Kyoto encyclopedia of genes and genomes; ko, KEGG orthology; CON, without seaweed extracts; AANC, *Amphiroa anceps*; ATAX, *Asparagopsis taxiformis*; CTEN, *Chondracanthus tenellus*; GELL, *Grateloupia elliptica*; GPAR, *Gracilaria parvispora*.

We found that enrichment of assimilatory sulfate reduction (M00176), which is an alternative H_2_ sink (75), was greater after GELL supplementation than after supplementation with the other extracts and CON. The end product of the sulfate reduction pathway, hydrogen sulfide can inhibit rumen methanogenic activity and consequently reduced CH_4_ production (76). Indeed, we found that the number of *Desulfovibrio* (sulfur metabolism and sulfate-reducing genus) was higher after ATAX supplementation than after CON supplementation (0.31 vs. 0.34%), although the difference was not significant. Additionally, we found that the genus *Dentirobacterium*, which occupies a very small proportion of the microbiota, was only observed after red seaweed extract supplementation, with ATAX showing the greatest enrichment (Table 4). *Denitrobacterium* species metabolize a variety of nitrocompounds in the rumen by oxidizing H_2_ or formate (77) and can consume lactate, which is an intermediate product, and H_2_ sink (78). Anderson et al. (79, 80) demonstrated that *Denitrobacterium detoxificans* added to the rumen culture primarily directly inhibit CH_4_ formation. One plausible explanation for this effect is that the *Denitrobacterium* genus may improve the ability of prokaryotes to utilize alternative H_2_ sinks, leading to a decline in the level of H_2_ available as a substrate for CH_4_ formation.

In summary, supplementation with red seaweed extracts can alter prokaryotic communities associated with propionate production, resulting in accelerated propionate production and reduced CH_4_ production. In addition, the relative abundances of *Methanobrevibacter* and total methanogens decreased following supplementation with red seaweed extracts, which was consistent with the decreased CH_4_ production. Although red seaweed extract supplementation may not substantially affect the overall microbiota and functional profiles, it affected the relative abundance of some prokaryotic microbiota and metabolic pathways. However, a large number of prokaryotes remain to be cultured, which is a limitation of this study. Further animal studies are needed to confirm the *in vitro* effects of red seaweed extract supplementation on fermentation characteristics, CH_4_ production, and microbiota.

## Data availability statement

The datasets presented in this study can be found in online repositories. The names of the repository/repositories and accession number(s) can be found at: https://www.ncbi.nlm.nih.gov/, PRJNA830647.

## Ethics statement

All experimental protocols were approved by the Animal Care and Use Committee (Approval ID: GNU-180130-A0007) of the Gyeongsang National University (Jinju, Gyeongsangnam-do, Korea). Written informed consent was obtained from the owners for the participation of their animals in this study.

## Author contributions

SSL, YC, and SJL designed the experiment. SJL provided funding. YC, SJL, HK, JE, and SJ conducted the experiment. YC and YL did the sequencing-based analysis. All the visualization of data and statistical analysis were performed by YC. HK, JE, SJ, and DB helped to revise the manuscript. JS provided rumen plasmid DNA for this study. LG, TP, DB, and SSL reviewed the manuscript, read, and approved the final manuscript. All authors contributed to the article and approved the submitted version.

## Funding

This study was supported by the National Institute of Animal Science, Ministry of Rural Development Administration, Republic of Korea (research project PJ01477803).

## Conflict of interest

The authors declare that the research was conducted in the absence of any commercial or financial relationships that could be construed as a potential conflict of interest.

## Publisher's note

All claims expressed in this article are solely those of the authors and do not necessarily represent those of their affiliated organizations, or those of the publisher, the editors and the reviewers. Any product that may be evaluated in this article, or claim that may be made by its manufacturer, is not guaranteed or endorsed by the publisher.

## Abbreviations

AANC: *Amphiroa* anceps
ATAX: *Asparagopsis taxiformis*
CTEN: *Chondracanthus tenellus*
GELL: *Grateloupia elliptica*
GPAR: *Gracilaria parvispora*
CE: Catechin equivalent
GAE: garlic acid equivalent
PCoA: principal coordinate analysis
LDA: linear discriminant analysis
LEfSe: linear discriminant analysis effect size
KEGG: Kyoto encyclopedia of genes and genomes.
