# Supplementary material for: Red seaweed extracts reduce methane production by altering rumen fermentation and microbial composition in vitro
Source: Front Vet Sci. 2022 Nov 16;9:985824. doi: 10.3389/fvets.2022.985824 (PMC9709288; doi:10.3389/fvets.2022.985824)
Supplement: Supplementary file 1 [file Date_Sheet_1.docx]

Supplementary Material

## Supplementary Tables

**Supplementary Table 1.** List of five red seaweed extracts provided from the MABIK in Korea

| Resource Number | Solvent | Scientific Name | Sampling Date | Sampling Sites |
| --- | --- | --- | --- | --- |
| MABIK NP30190008 | 70% EtOH | *Amphiroa anceps* | 2017.07.21 | Jeju-do |
| MABIK NP30150028 | 70% EtOH | *Asparagopsis taxiformis* | 2011.04.23 | Jeju-do |
| MABIK NP30160019 | 70% EtOH | *Chondracanthus tenellus* | 2016.06.30 | Jeollanam-do |
| MABIK NP30160020 | 70% EtOH | *Grateloupia elliptica* | 2016.06.30 | Jeollanam-do |
| MABIK NP30150011 | 70% EtOH | *Gracilaria parvispora* | 2011.08.24 | Jeju-do |

MABIK: marine biodiversity institute of Korea

**Supplementary Table 2.** Primers (F = forward, R = reverse) for real-time PCR assay

| Target species | Primer sequence (5′→3′) | Size (bp)^a^ | Reference |
| --- | --- | --- | --- |
| Total bacteria | F: CGGCAACGAGCGCAACCC | 130 | Denman and McSweeney (2006) |
|  | R: CCATTGTAGCACGTGTGTAGCC |  |  |
| Ciliate protozoa | F: GCTTTCGWTGGTAGTGTATT | 223 | Sylvester et al. (2004) |
|  | R: CTTGCCCTCYAATCGTWCT |  |  |
| Fungi | F: GAGGAAGTAAAAGTCGTAACAAGGTTTC | 120 | Denman and McSweeney (2006) |
|  | R: CAAATTCACAAAGGGTAGGATGATT |  |  |
| Total methanogens | F: CCGGAGATGGAACCTGAGAC | ~160 | Zhou et al. (2009) |
|  | R: CGGTCTTGCCCAGCTCTTATTC |  |  |

^a^bp, base pair. F: forward; R: revers

**Supplementary Table 3.** Number of detected functional features of the microbiota on in vitro using by PICURSt2

| Measurement | CON | Treatments | | | | | SEM | *P*-value |
| --- | --- | --- | --- | --- | --- | --- | --- | --- |
|  |  | AANC | ATAX | CTEN | GELL | GPAR |  |  |
| Functional features | | | | | | | | |
| COG | 3623 | 3644 | 3577 | 3630 | 3636 | 3639 | 36 | 0.80 |
| PFAM | 5261 | 5288 | 5218 | 5271 | 5269 | 5292 | 59 | 0.95 |
| EC | 1465 | 1482 | 1456 | 1468 | 1482 | 1475 | 19 | 0.92 |
| MetaCyc pathways | 295 | 300 | 294 | 297 | 299 | 297 | 5 | 0.93 |
| KEGG orthologs | 4263 | 4330 | 4212 | 4278 | 4326 | 4309 | 79 | 0.89 |
| KEGG modules | 258 | 256 | 254 | 255 | 257 | 258 | 2 | 0.62 |
| KEGG pathways | 132 | 130 | 131 | 131 | 130 | 131 | 1 | 0.40 |

SEM: standard error of the mean; amplicon sequence variant; COG: clusters of orthologous genes; PFAM: protein families; EC: enzyme classification; KEGG: kyoto encyclopedia of genes and genomes; CON**:** without seaweed extracts; AANC: *Amphiroa anceps*; ATAX: *Asparagopsis taxiformis*; CTEN: *Chondracanthus tenellus*; GELL: *Grateloupia elliptica*; GPAR: *Gracilaria parvispora*.

## Supplementary Figures

**
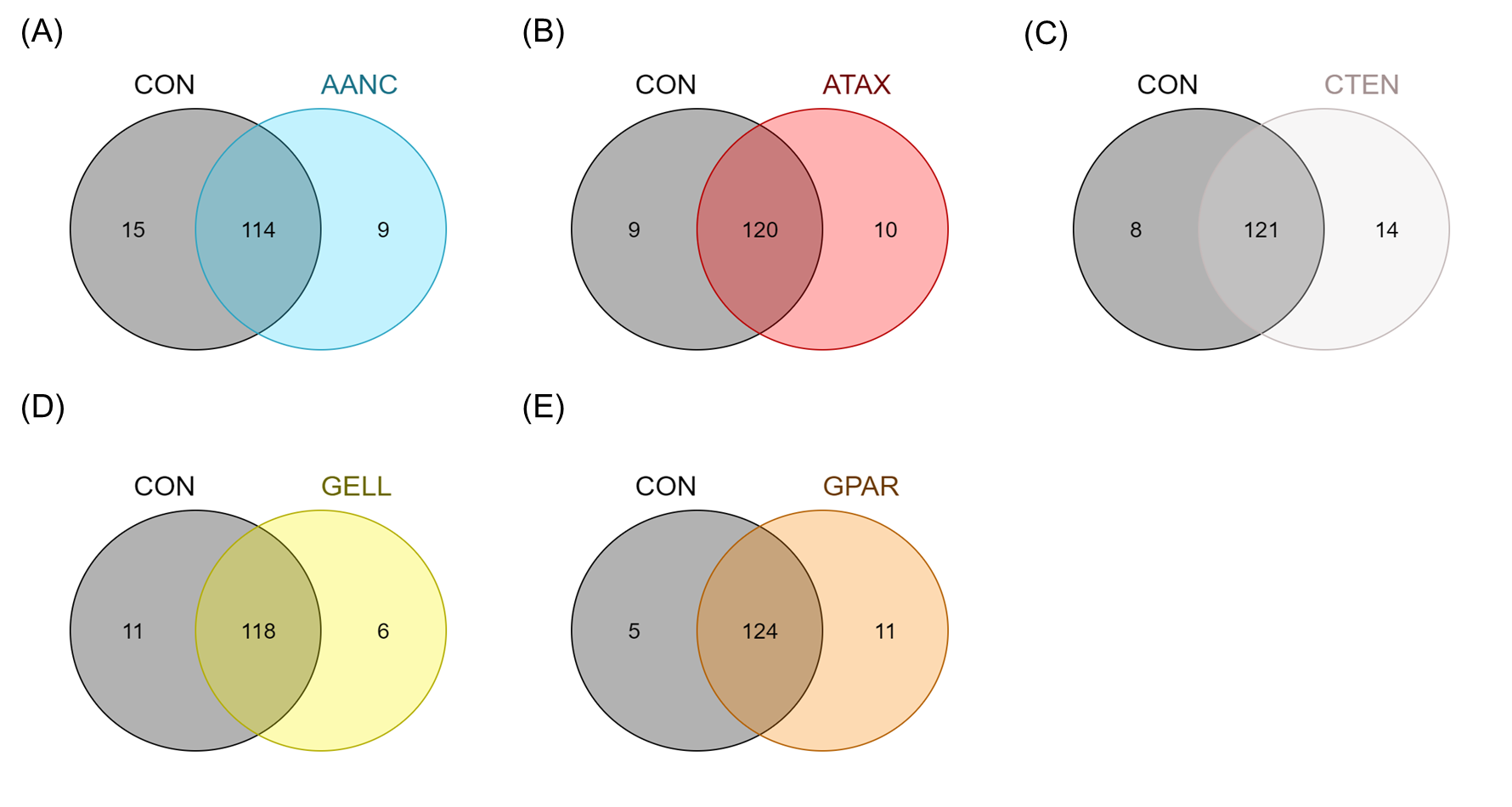
**

**Supplementary Figure 1.** Number of bacterial and archaeal genera which were differentially observed by red seaweed extracts. The number of shared or exclusively found genera or functional features among the corresponded CON and red seaweed extracts were shown in Venn diagram. CON: without seaweed extracts; AANC: *Amphiroa anceps*; ATAX: *Asparagopsis taxiformis*; CTEN: *Chondracanthus tenellus*; GELL: *Grateloupia elliptica*; GPAR: *Gracilaria parvispora*.


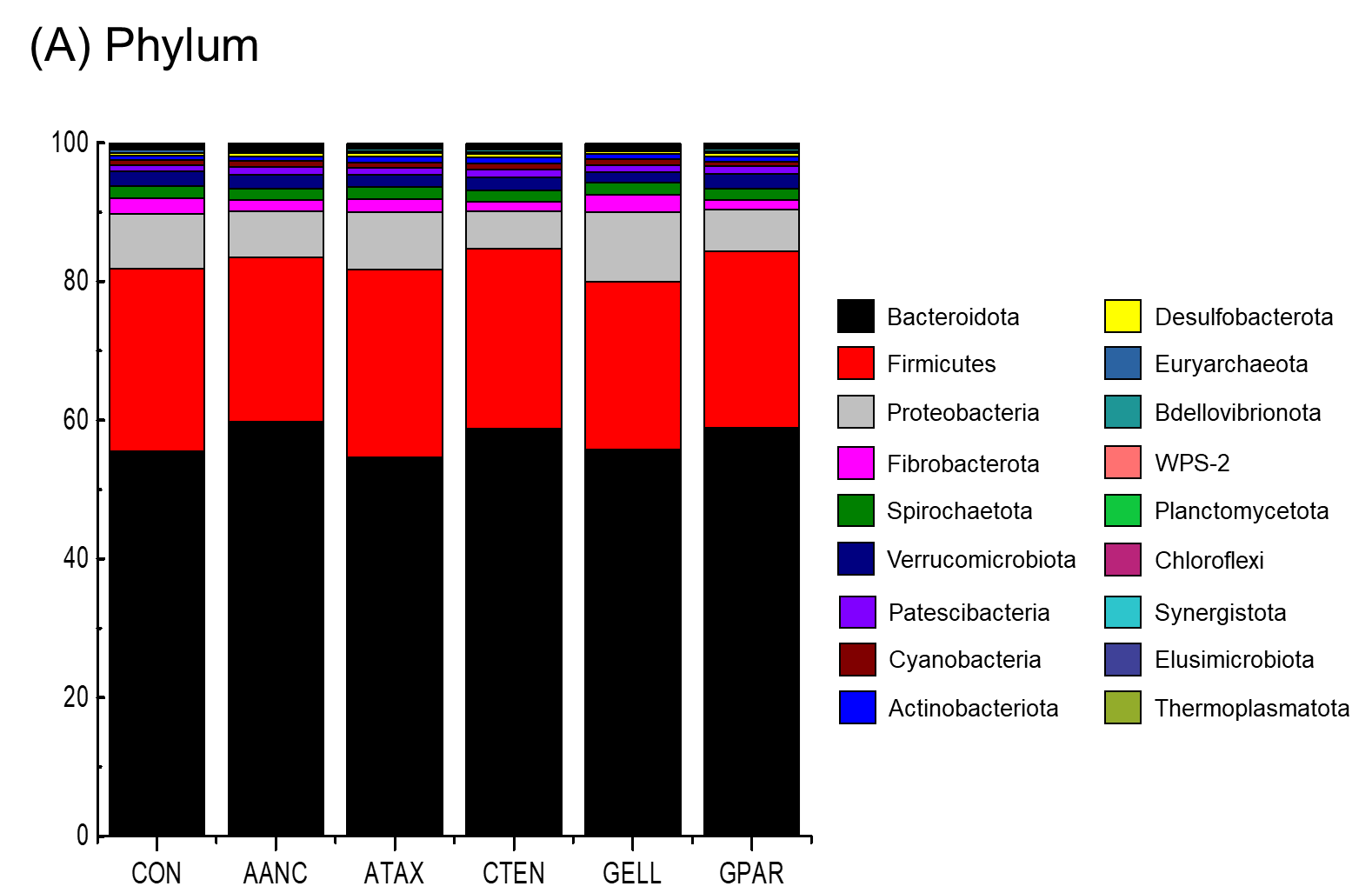


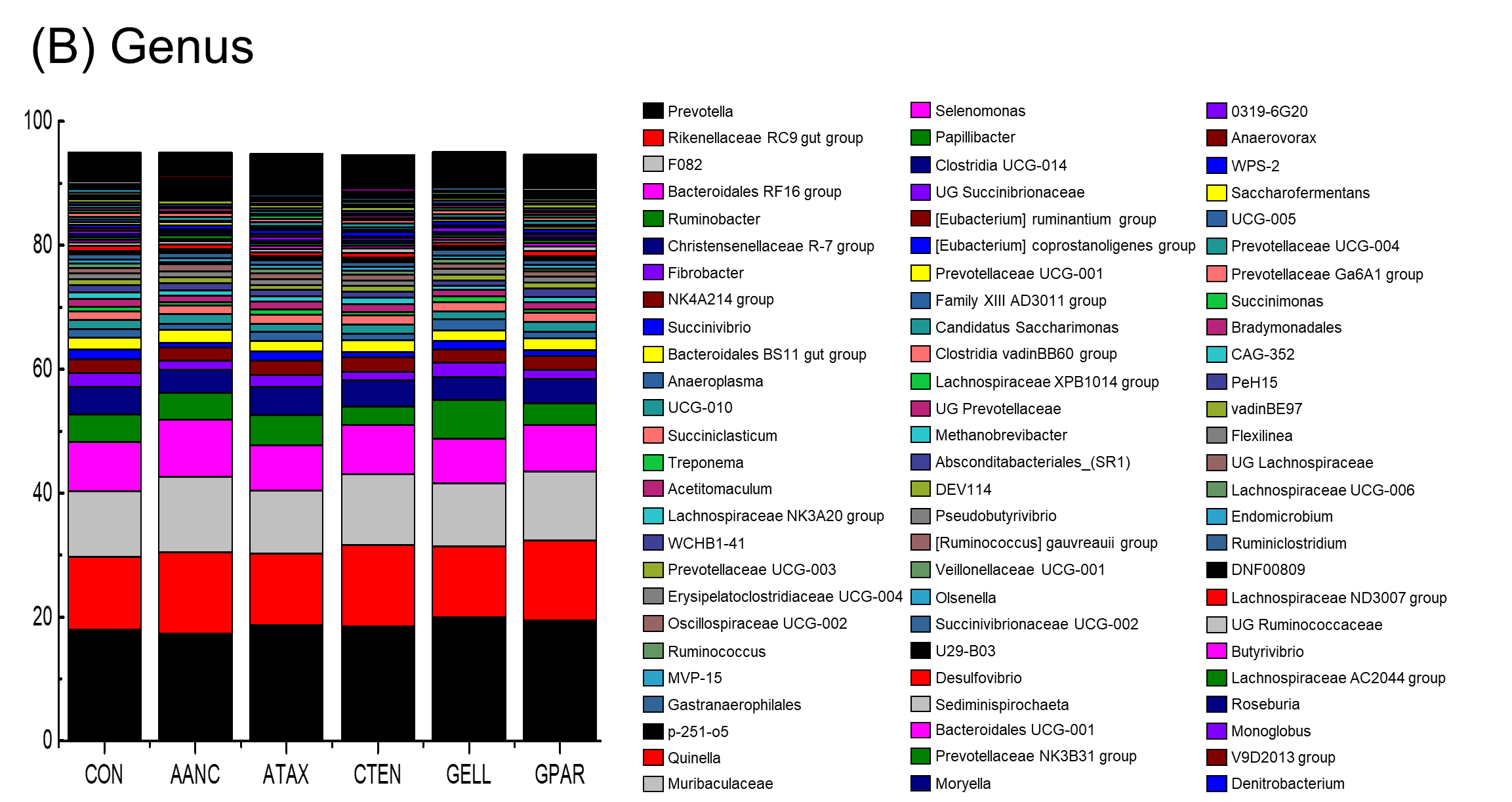


**Supplementary Figure 2.** Taxonomic profiles of the relative of phylum-level (A) and genus-level (B) abundances of microbiota in CON and red seaweed extracts, with a classification criterion of > 0.1% of the phylum and genus-level of the total sequences (after 24 h of incubation). CON**:** without seaweed extracts; AANC: *Amphiroa anceps*; ATAX: *Asparagopsis taxiformis*; CTEN: *Chondracanthus tenellus*; GELL: *Grateloupia elliptica*; GPAR: *Gracilaria parvispora*; UCG: uncultured genus-level group; UG: unclassified genus.


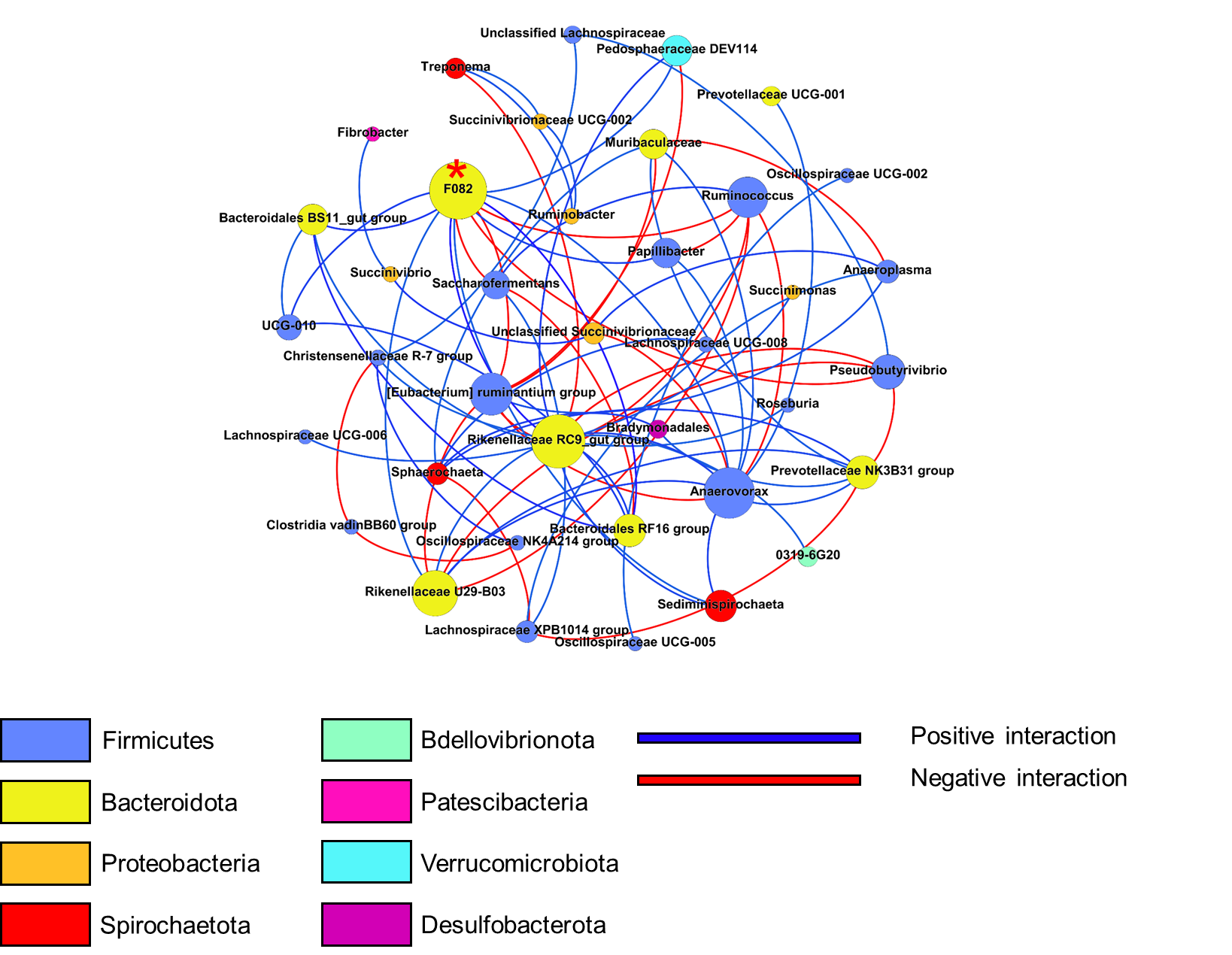


**Supplementary Figure 3.** Co-occurrence network inferred from the SparCC algorithm applied to major prokaryotic genera (each representing > 0.1% at least one of treatments) from red seaweed extract supplementation. Only strong significant correlation coefficients (|*r*| > 0.7, *P* < 0.05) were shown on the network plot. Blue and red lines indicate positive (co-occurrence) and negative (mutual-exclusive) interaction, respectively. Color legend showed the belonged phylum of each node. Red asterisk indicates best centrality genus based on degree, authority and eigenvector centrality.
